# Metabolites of the alkyl pyrrolidone solvents NMP and NEP in 24-h urine samples of the German Environmental Specimen Bank from 1991 to 2014

**DOI:** 10.1007/s00420-018-1347-y

**Published:** 2018-08-22

**Authors:** Nadin Ulrich, Daniel Bury, Holger M. Koch, Maria Rüther, Till Weber, Heiko-Udo Käfferlein, Tobias Weiss, Thomas Brüning, Marike Kolossa-Gehring

**Affiliations:** 1 Institute for Prevention and Occupational Medicine of the German Social Accident Insurance, Institute of the Ruhr-Universität Bochum (IPA), Bürkle-de-la-Camp Platz 1, 44789 Bochum, Germany; 2 Department of Analytical Environmental Chemistry, Helmholtz-Centre for Environmental Research-UFZ, Permoserstr. 15, 04318 Leipzig, Germany; 3 German Environment Agency (UBA), Corrensplatz 1, 14195 Berlin, Germany

**Keywords:** Human biomonitoring, *N*-Methyl-2-pyrrolidone (NMP), *N*-Ethyl-2-pyrrolidone (NEP), German Environmental Specimen Bank (ESB), Exposure assessment, Urinary metabolites

## Abstract

**Purpose:**

The aim of this study was to get a first overview of the exposure to the solvents and reproductive toxicants *N*-methyl-2-pyrrolidone (NMP) and *N*-ethyl-2-pyrrolidone (NEP) in Germany. NMP and NEP metabolite concentrations were determined in 540 24-h urine samples of the German Environmental Specimen Bank collected from 1991 to 2014. With these data we were able to investigate NMP/NEP exposures over time and to evaluate associated risks.

**Methods:**

NMP metabolites 5-hydroxy-*N*-methyl-2-pyrrolidone (5-HNMP) and 2-hydroxy-*N*-methylsuccinimide (2-HMSI) and NEP metabolites 5-hydroxy-*N*-ethyl-2-pyrrolidone (5-HNEP) and 2-hydroxy-*N*-ethylsuccinimide (2-HESI) were determined by stable isotope dilution analysis using solid phase extraction followed by derivatization (silylation) and GC–EI–MS/MS.

**Results:**

We were able to quantify 5-HNMP and 2-HMSI in 98.0 and 99.6% and 5-HNEP and 2-HESI in 34.8 and 75.7% of the samples. Metabolite concentrations were rather steady over the timeframe investigated, even for NEP which has been introduced as an NMP substitute only in the last decade. Calculated median daily intakes in 2014 were 2.7 µg/kg bw/day for NMP and 1.1 µg/kg bw/day for NEP. For the combined risk assessment of NMP and NEP exposure, the hazard index based on the human biomonitoring assessment I values (HBM I values) was less than 0.1.

**Conclusions:**

Based on the investigated subpopulation of the German population, individual and combined NMP and NEP exposures were within acceptable ranges in the investigated timeframe. Sources of NEP exposure in the 90s and 00s remain elusive.

**Electronic supplementary material:**

The online version of this article (10.1007/s00420-018-1347-y) contains supplementary material, which is available to authorized users.

## Introduction

*N*-Methyl-2-pyrrolidone (NMP, CAS 872-50-4) and*N*-ethyl-2-pyrrolidone (NEP, CAS 2687-91-4) are dipolar aprotic solvents with high solvent power and water solubility. In Europe 18,000 tons of NMP are produced each year (ECHA 2013). It is estimated, that the overall produced and imported amount of NMP on the European market is between 10,000 and 100,000 tons per year (ECHA 2016a). For NEP it is reported that between 1000 and 10,000 tons are produced and/or imported on the European market (ECHA 2016b).

NMP is extensively used as solvent in the production of polymers, petrochemicals, coating products, waterborne paints, electronics, semiconductor materials, and batteries. In addition, it is used as cleaning agent to remove plastics, oil, glue, grease, and paints. Minor uses are in pharmaceutical industry (process solvent and enhancer for transdermal delivery of drugs) and agriculture (component in selected pesticide formulations). Different consumer products may include NMP up to an amount of 5%, such as printer inks, toners, coatings, cleaners, and ink (ECHA 2013). Due to similar physicochemical properties, NEP is an equally functional solvent and cleaner. However, little is known about the application fields where NEP is currently used. Main industrial sectors have been reported of being similar to those of NMP (ECHA 2011, 2016b).

In 2009 NMP was classified as a reproductive toxicant Cat. 1B (H360), with labeling requirements in mixtures containing ≥ 5% NMP [Commission Regulation (EC) No 790/2009]. The classification is based on studies where dose-related decreases have been observed in fetal body weights of rats after oral and inhalation exposure and at levels below maternal toxicity (Saillenfait et al. 2002, 2003, 2007a). Further fetal malformations were found at maternally toxic doses. In 2011 NMP was included in ECHA´s candidate list for authorisation as a substance of very high concern (SVHC). Due to these developments, NMP has increasingly been substituted by NEP (WO-Patent WO/2005/090447; US-Patent US 7,994,350 B2). However, NEP has been shown to possess a toxicological profile similar to NMP. Developmental effects (reduction of fetal weights and a pattern of malformation) were observed in rat studies (Saillenfait et al. 2007b) at comparable doses to NMP. In 2013 (effective January 2015), NEP has also been classified as a reproductive toxicant Cat. 1B H360D [Commission Regulation (EU) No 317/2014; Commission Regulation (EU) No 944/2013]. Today, consumer products with an NMP or NEP content ≥ 0.3% have to be labeled as category 1B reproductive toxicant according to the Globally Harmonized System of Classification and Labelling of Chemicals (GHS) [Regulation (EC) No 1272/2008; Commission Regulation (EU) No 944/2013]. Furthermore, in Europe, the use of reproductive toxicants (Cat 1A and 1B) in cosmetics is prohibited under Article 15(2) of the Cosmetics Regulation 1223/2009.

Because of the ubiquitous occurrence and toxic properties it was decided in the cooperation project between the German Federal Ministry for the Environment, Nature Conservation and Nuclear Safety (BMU) and the Verband der Chemischen Industrie e.V. (German Chemical Industry Association—VCI) (Kolossa-Gehring et al. 2017) to develop an analytical method for biomonitoring of NMP and NEP metabolites and apply it in a population study.

Both, NMP and NEP can be absorbed by skin, inhalation or by ingestion. Human NMP metabolism has been previously studied in detail (Åkesson and Jönsson 1997; Åkesson et al. 2004; Bader et al. 2007, 2008). Metabolites result from oxidation by cytochrome P450 enzymes. First, 5-hydroxy-*N*-methyl-2-pyrrolidone (5-HNMP) is formed, which is further oxidized to *N*-methylsuccinimide (MSI) and 2-hydroxy-*N*-methylsuccinimide (2-HMSI). 5-HNMP and 2-HMSI are the main metabolites which can be detected in urine. In example, 43.8% of NMP are excreted as 5-HNMP and 19.7% as 2-HMSI with elimination half-times of ~ 4 h for 5-HNMP and ~ 17 h for 2-HMSI. Investigations on NEP metabolism in humans is limited (Koch et al. 2014). However, analogous major urinary metabolites, 5-hydroxy-*N*-ethyl-2-pyrrolidone (5-HNEP) and 2-hydroxy-*N*-ethylsuccinimide (2-HESI), have been identified after oral dosages. Here, 28.9% of the dose are excreted as 5-HNEP and 21.6% as 2-HESI. The elimination half-times are ~ 7 h and ~ 22 h, respectively.

In 2015, HBM values (Human Biomonitoring assessment values) for NMP and NEP were published by the German Human Biomonitoring Commission (HBM Commission) (Bekanntmachung des Umweltbundesamtes 2015; Stellungnahme der Kommission “Humanbiomonitoring” des Umweltbundesamtes 2015). These HBM values are derived on the basis of toxicological studies and allow a risk assessment based on the sum of the respective major urinary metabolite levels (5-HNMP and 2-HMSI in case of NMP and 5-HNEP and 2-HESI in case of NEP). For adults the HBM I value of NMP, a level below which there is no risk for adverse health effects and no need for action, is currently 15 mg/L for the sum of 5-HNMP and 2-HMSI. The same is true for NEP and its metabolites 5-HNEP and 2-HESI. The HBM II value of NMP, a level above which there is an increased risk for adverse health effects and an acute need for exposure reduction measures (action level), is 50 mg/L for the sum of both NMP metabolites; whereas it is 40 mg/L for the sum of NEP metabolites (Apel et al. 2017).

Due to the widespread uses of NMP and NEP, an omnipresent exposure of the general population has to be assumed. In a pilot study on 56 individuals from the general German population we detected NMP metabolites in 98% and NEP metabolites in 48% of the urine samples (Schindler et al. 2012). As a next step to confirm this data we studied 24-h urine samples of the German Environmental Specimen Bank (ESB) (Wiesmüller et al. 2007; Kolossa-Gehring et al. 2012). The ESB collects human specimens (urine, whole blood, and blood plasma) since 1981. These continuously collected and biobanked 24-h urine samples enabled us to retrospectively investigate exposures to NMP and NEP and to derive and investigate time trends of exposure. In addition, we calculated daily exposures and daily intakes (DIs) based on the known metabolic conversion factors for NMP and NEP. Finally, the comparison of metabolite concentrations with health-based guidance values (such as HBM values) allowed us to carry out a cumulative risk assessment. Several studies to identify time trends in body burdens, DI and cumulative risk assessment have already been completed, e.g., on phthalates (Koch et al. 2017) and phthalate substitutes such as DINCH (Schütze et al. 2014) and DPHP (Schütze et al. 2015), BPA (Koch et al. 2012), Glyphosate (Conrad et al. 2017), PFAAs and PFSAs (Schröter-Kermani et al. 2013) and several parabens (Moos et al. 2015). Here we add additional data on NMP and NEP.

## Materials and methods

### Subjects and human specimens

Urine samples were collected by the ESB in the years 1991, 1995, 1999, 2003, 2006, 2008, 2010, 2012, and 2014. The ESB is under responsibility of the German Federal Ministry for Environment, Nature Conservation and Nuclear Safety (BMU) and the German Environment Agency (UBA) (Wiesmüller et al. 2007). Study protocol of human specimen sampling has been reviewed and approved by the ethics committee of the Medical Association Westfalen-Lippe and the Medical Faculty of the University of Münster and since 2012 by the ethics committee of the Medical Association Saarland.

Overall 540 24-h urine samples were investigated. For each year 60 samples were included (30 male/30 female). Samples were from students between 20 and 30 years of age. Sampling location was the area of Münster. Demographic data (e.g., age, gender, body height) was collected by a standardized self-reported questionnaire. Details can be found in Table 1. Until 2011, urinary creatinine was measured photometrically according to Jaffe (Boehringer Mannheim, MPR3 Creatinine testkit, 124 192). Since 2012 urine samples have been analyzed using a colorimetric determination based on the Jaffe reaction using a cobas c111 analyzer (Roche, 04 777 433 001) (Lermen et al. 2014). Samples were blinded by the ESB before shipment and analysis at IPA laboratories, Bochum, Germany.

**Table 1:** Detailed description of the samples from the German Environmental Specimen Bank (ESB)

Sampling year	Subjects (male/female)	Age in years median (range)	Body weight (kg) median (range)	24 h volume (mL) median (range)	Creatinine content (g/L) median (range)
1991	60 (30/30)	24 (22–29)	66.5 (49–91)	1200 (400–2800)	1.213 (0.402–3.146)
1995	60 (30/30)	24 (20–29)	70 (52–100)	1530 (450–2600)	1.091 (0.400-2.748)
1999	60 (30/30)	24 (21–29)	67 (46–175)	1420 (550–4000)	1.022 (0.275–2.656)
2003	60 (30/30)	23 (20–28)	69 (49–105)	1770 (410–3500)	0.905 (0.230–3.410)
2006	60 (30/30)	24 (20–29)	69.5 (47–95)	1835 (719–4250)	0.746 (0.290–2.108)
2008	60 (30/30)	23,5 (20–29)	68.5 (47–102)	1996 (855–5460)	0.722 (0.150–2.150)
2010	60 (30/30)	23 (20–28)	72.5 (50–101)	1864 (749–5168)	0.710 (0.281–2.188)
2012	60 (30/30)	24 (20–30)	70 (48–105)	2121 (574–3027)	0.612 (0.205–2.181)
2014	60 (30/30)	23 (20–29)	68.5 (49–95)	1886 (844–3050)	0.714 (0.189–2.048)
In total	540 (270/270)	24 (20–30)	69 (46–175)	1770 (400–5460)	0.837 (0.150–3.410)
Male	270	24 (20–30)	76 (52–175)	1800 (410–5168)	0.998 (0.189–3.410)
Female	270	23 (20–30)	60 (46–105)	1710 (400–5460)	0.698 (0.150–2.748)

### Chemicals

All reagents and chemicals were purchased and used as previously described (Schindler et al. 2012). In particular, (±)-5-Hydroxy-*N*-ethyl-2-pyrrolidone (5-HNEP), 2-hydroxy-*N*-ethylsuccinimide (2-HESI) and the ethyl chain deuterium-labeled analogues (±)-5-hydroxy-*N*-ethyl-2-pyrrolidone-d5 (5-HNEP-d5) and (±)-2-hydroxy-*N*-ethylsuccinimide-d5 (2-HESI-d5) were synthesized and characterized. The chemical purity of the aforementioned compounds was > 97%. The deuterated internal standards had no detectable impurities of non-labeled analogues.

### Sample preparation and analysis

Quantitative analysis of NMP and NEP metabolites in human urine samples was carried out in one analytical run based on a previously described method (Schindler et al. 2012) and included the NMP metabolites 5-HNMP and 2-HMSI and the NEP metabolites 5-HNEP and 2-HESI. This method, originally developed for occupational exposure has been modified to achieve increased sensitivities to enable the quantification of metabolite concentrations in samples from the general population. This was achieved using tandem mass spectrometry instead of simple mass selective detection (MSD) and a slightly modified sample preparation procedure as follows:

200 µL internal standard (*c* = 5 mg/L), 200 µL water, and 200 µL 1% acetic acid were added to 600 µL urine. After homogenization, 1000 µL of this solution were loaded onto solid phase extraction cartridges [Isolute ENV + (hydroxylated polystyrene-divinylbenzene co-polymer), 100 mg, 1 mL cartridges, Biotage AB, Uppsala, Sweden], preconditioned with 2 × 500 µL ethyl acetate/ethanol (4:1 v/v), 2 × 500 µL methanol, and 4 × 500 µL 1% acetic acid. After washing with 250 µL 1% acetic acid and 750 µL water, the analytes were eluted with 1750 µL ethyl acetate/ethanol (4:1 v/v). Samples were dried under a gentle stream of nitrogen, then dissolved in 200 µL acetonitrile, and again dried under nitrogen. After adding 30 µL pyridine and 30 µL *N*-*tert*-butyldimethylsilyl-*N*-methyltrifluoroacetamide for derivatization (110 °C, 60 min), the samples were finally diluted with 50 µL ethyl acetate and analyzed by GC-EI-MS/MS.

GC–EI–MS/MS (7890B GC System, 7000C GC/MS Triple Quad, Agilent Technologies, Waldbronn, Germany) analysis was performed using an Rtx^®^-35w/Integra-Guard^®^ capillary column (60 m, 10 m pre-column, 0.25 mm internal diameter, 0.25 µm film thickness, Restek, Bellefonte, USA). Helium 6.0 (1 mL/min) was used as carrier gas, injection volume was 1 µL. Injection was performed in pulsed splitless mode at an injector temperature of 280 °C. The temperature program was as follows: 90 °C (5 min); 10°C/min to 200 °C (5 min); 30°C/min to 280 °C (6 min). Transfer line, ion source, and quadrupole temperatures were set to 280, 230, and 150 °C, respectively. Collision gas (nitrogen 5.0) flow rate was 1.5 mL/min and quench gas flow rate (Helium 6.0) was 2.25 mL/min. Quantification (quantifier) and confirmation (qualifier) of the respective analytes were done in EI MRM mode (70 eV, Table 2).

**Table 2:** Retention times (RT), MRM conditions (quantifier, qualifier), and collision energies

Analyte	RT (min)	Quantifier (*m*/*z*)	Collision energy (eV)	Qualifier (*m*/*z*)	Collision energy (eV)
2-HMSI	19.9	186.0 → 144.0	10	186.0 → 129.0	10
2-HESI	20.3	200.0 → 158.0	5	200.0 → 172.0	7.5
5-HNMP	20.4	172.0 → 98.0	15	172.0 → 126.0	15
5-HNEP	20.9	186.0 → 112.0	5	186.0 → 140.0	15
2-HMSI-*d*_*3*_	19.9	189.0 → 129.0	12.5	189.0 → 119.0	12.5
5-HNMP-*d*_*4*_	20.3	176.0 → 102.0	15	176.0 → 128.0	15
2-HESI-*d*_*5*_	20.1	205.0 → 163.0	15	205.0 → 177.0	15
5-HNEP-*d*_*5*_	20.8	191.0 → 117.0	15	191.0 → 145.0	15

LOQs were determined according to a signal to noise ratio of nine. The LOQ for 2-HMSI and 2-HESI was 2 µg/L and for 5-HNMP and 5-HNEP it was 2.5 µg/L. Information about within-series and day-to-day imprecision are given in Table 3. In-house quality control measures (Q low, Q high) were included in each series of samples analyzed. Both Q low and Q high-quality standards were also used in method validation (Table 3). Further, interlaboratory comparison was assured by successful participation in the external quality assessment scheme G-Equas (http://www.g-equas.de) for NMP metabolites.

**Table 3:** Within-series and day-to-day imprecision for NMP and NEP metabolites, concentrations of spiked urine samples (Q low and Q high) are given in brackets

Analyte	Within-series imprecision		Day-to-day imprecision	
	Q low	Q high	Q low	Q high
2-HMSI	6.6% (47 µg/L)	2.1% (280 µg/L)	12.4% (52 µg/L)	10.4% (310 µg/L)
2-HESI	8.5% (56 µg/L)	5.1% (330 µg/L)	9.0% (58 µg/L)	10.7% (340 µg/L)
5-HNMP	8.3% (49 µg/L)	2.9% (280 µg/L)	9.8% (51 µg/L)	7.0% (280 µg/L)
5-HNEP	8.4% (88 µg/L)	2.8% (500 µg/L)	12.3% (87 µg/L)	5.7% (480 µg/L)

### Statistics, hazard index and daily intake calculation

Statistical analysis was performed using IBM SPSS Statistics 22 (2013). Boxplots were generated with OriginPro2015G (OriginLab Corporation). Values below LOQ were set as LOQ/2 (1.0 µg/L for 2-HMSI and 2-HESI, 1.3 µg/L for 5-HNMP and 5-HNEP), values below LOD (limit of detection) were set as LOQ/4 (0.5 µg/L for 2-HMSI and 2-HESI, 0.6 µg/L for 5-HNMP and 5-HNEP). To investigate possible associations between creatinine adjusted metabolite levels and gender, the Mann-Whitney U test was applied. Jonckheere–Terpstra test was applied to analyze time trends.

For cumulative risk assessment of NMP and NEP the hazard index (HI) was calculated as the sum of hazard quotients (HQ) for NMP and NEP (Teuschler and Hertzberg 1995; Kortenkamp and Faust 2010; Søeborg et al. 2012). HQs were calculated as the ratio between the sum of 2-HMSI and 5-HNMP (for NMP) or 2-HESI and 5-HNEP (for NEP), respectively, and HBM I as acceptable levels. The daily intake (DI) in µg/kg bw/day was calculated according to Schütze et al. (2014) using Eq. (1) by the concentrations of both respective metabolites c_m1_ and c_m2_ (5-HNMP and 2-HMSI or 5-HNEP and 2-HESI) in µg/L and their respective molar masses M(m1) and M(m2) in g/mol, the molar masses of NMP or NEP M(compound) in g/mol, the 24-h urine volume in L/d, the urinary excretion factors f_UE_(m1) and f_UE_(m2) of the respective metabolites (see Åkesson et al. 1997; Koch et al. 2014), and the body weight of the test persons in kg. The DI were calculated for each sample, and the median DIs and 95th percentiles were calculated for each investigated year.

1 $${\text{D}}{{\text{I}}_{{\text{compound}}}}=\frac{{\left( {{\raise0.7ex\hbox{${c\left( {m1} \right)}$} \!\mathord{\left/ {\vphantom {{c\left( {m1} \right)} {M\left( {m1} \right)}}}\right.\kern-0pt}\!\lower0.7ex\hbox{${M\left( {m1} \right)}$}}+{\raise0.7ex\hbox{${c\left( {m2} \right)}$} \!\mathord{\left/ {\vphantom {{c\left( {m2} \right)} {M(m2)}}}\right.\kern-0pt}\!\lower0.7ex\hbox{${M(m2)}$}}} \right) \times M({\text{compound}}) \times {V_{24~{\text{h~urine}}}}}}{{\left( {{f_{{\text{UE}}}}(m1)+{f_{{\text{UE}}}}(m2)} \right) \times {\text{b}}{{\text{w}}_{{\text{proband}}}}}}.$$

## Results and discussion

The urinary metabolite levels of NMP and NEP are presented in Table 4 (µg/g creatinine; SI 1: µg/L; for correlations 5-HNMP/2-HMSI and 5-HNEP/2-HESI see SI 2). NMP metabolites were above LOQ in 98% (5-HNMP) and 99.6% (2-HMSI) of the samples. The NEP detection rates were lower, with 34.8% (5-HNEP) and 75.7% (2-HESI) of the samples above LOQ. The detection rates clearly indicate that the investigated population is exposed to both NMP and NEP. Median NMP metabolite levels (5-HNMP 30.3 µg/L, 2-HMSI 38.8 µg/L) were generally higher than NEP metabolite levels (5-HNEP < LOQ, 2-HESI 6.1 µg/L) while 95th percentiles were higher for NEP (5-HNEP: 212 µg/L, 2-HESI: 230 µg/L) than NMP (5-HNMP: 98.1 µg/L, 2-HMSI: 100 µg/L). Metabolite concentrations were in the same range as those from a pilot population (Schindler et al. 2012) and reference populations (Meier et al. 2013; Koslitz et al. 2014) of occupationally non-exposed individuals.

**Table 4:** Creatinine adjusted concentrations of the respective NMP and NEP metabolites for all years

Concentration (µg/g creatinine)	NMP		NEP	
	5-HNMP	2-HMSI	5-HNEP	2-HESI
All samples				
Median	37.3	45.3	< LOQ	8.0
Geometric mean	34.7	45.3	3.4	10.4
95th percentile	113	111	279	338
Min	< LOQ	< LOQ	< LOQ	< LOQ
Max	386	538	1061	1019
Male				
Median	32.7	39.2	<LOQ	7.1
Geometric mean	28.4	41.2	2.5	8.2
95th percentile	100	106	193	205
Min	< LOQ	7.1	< LOQ	< LOQ
Max	386	267	823	862
Female				
Median	43.4	50.7	< LOQ	10.3
Geometric mean	42.4	49.9	4.7	13.3
95th percentile	115	117	329	403
Min	< LOQ	< LOQ	< LOQ	< LOQ
Max	239	538	1061	1019

Neither NMP nor NEP metabolite levels exceeded HBM I values. The maximum concentration of the sum of the NMP metabolites observed in this study was 1,013 µg/L (1.01 mg/L), being a factor of 15 below the HBM-I value for adults (15 mg/L) and a factor of 49 below the respective HBM-II value (50 mg/L). The maximum concentration of the sum of NEP metabolites was 1,312 µg/L (1.31 mg/L), being a factor of 11 and 30 below the HBM-I (15 mg/L) and HBM-II (40 mg/L) levels. These HBM values apply to the evaluation of a single substance. Due to the very similar toxicological profiles of NMP and NEP, a mixed exposure to both substances has to be taken into account in risk assessment [Stellungnahme der Kommission “Humanbiomonitoring” des Umweltbundesamtes (2015)]. The hazard index (HI), based on the individual HBM I values of NMP and NEP taking into account co-exposure was 0.09 at maximum [maximum HQs: 0.07 (NMP) and 0.09 (NEP)] with a median of 0.01. An HI of > 1 would indicate a combined exceedance of the HBM I values. Consequently, the results of this study do not raise toxicological concerns towards the level of combined NMP and NEP exposure of the population within the investigated timeframe (1991–2014).

**Fig. 1:**
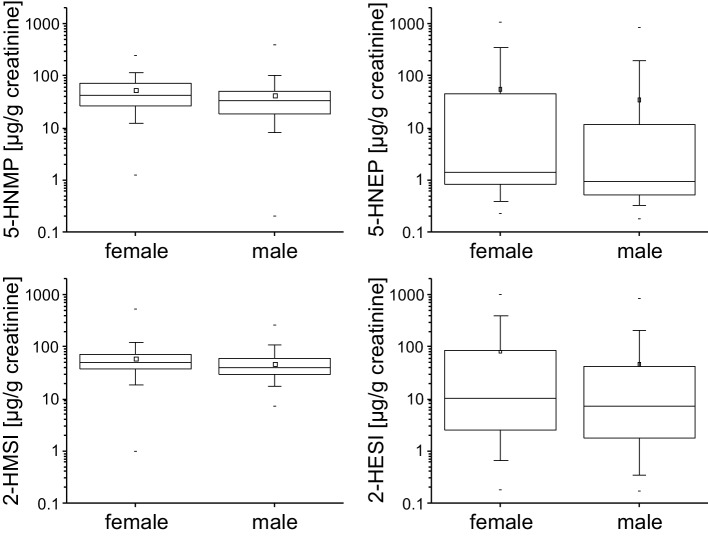
Boxplots of the metabolites 5-HNMP, 5-HNEP, 2-HMSI, and 2-HESI for males and females. The concentrations are given in [µg/g creatinine], the arithmetic mean is indicated by the squared dot, the boxes show the 25th, 50th, and 75th percentile and the whiskers the 5th and 95th percentile, respectively. The minimum and maximum concentrations are indicated by a dash

For all four metabolites, creatinine adjusted concentrations were slightly higher in females than in males (Fig. 1). These differences were statistically significant for 2-HMSI (*p* < 0.001), 5-HNMP (*p* < 0.001), and 2-HESI (*p* = 0.012). Due to the lower detection rates, 5-HNEP was excluded from this and further statistical analyses. Potential sources of exposure of the general population to NMP and NEP cannot clearly be identified, although it has been reported that NMP exposure may arise from the use of pharmaceutical or cosmetic products (Bekanntmachung des Umweltbundesamtes 2015). Furthermore, NMP is applied as additive in inks, toners and also cleaning agents (ECHA 2013). Therefore, further studies will be needed to identify exposure sources of NMP and NEP in the general population.

**Fig. 2:**
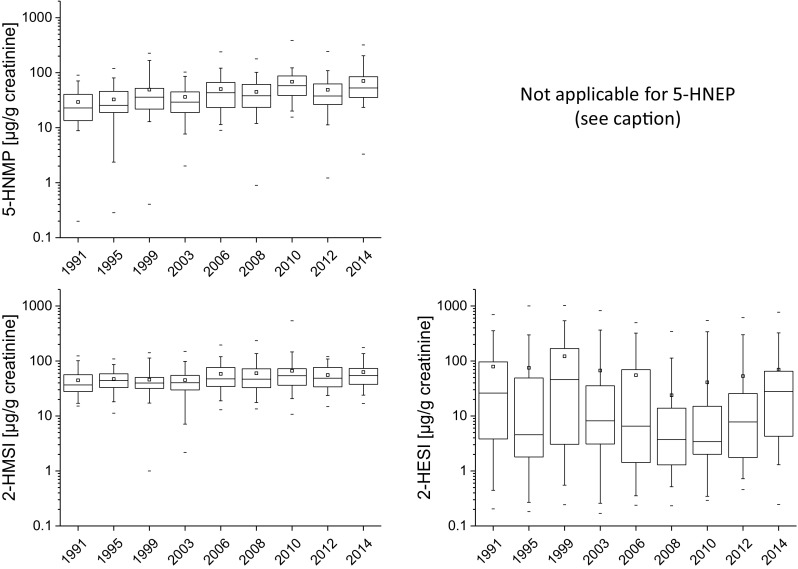
Boxplots of the metabolites 5-HNMP, 5-HNEP, 2-HMSI, and 2-HESI for the investigated years. The concentrations are given in (µg/g creatinine), the average is indicated by the squared dot, the boxes show the 25th, 50th, and 75th percentile and the whiskers the 5th and 95th percentile, respectively. The minimum and maximum concentrations are indicated by a dash. 5-HNEP was excluded due to most concentrations (in µg/L) being below LOQ

The urinary alkyl pyrrolidone metabolite concentrations for the investigated years between 1991 and 2014 are presented in Table 5 (and SI 3) and Fig. 2 (and SI 4). The median NMP metabolite concentrations vary within rather tight boundaries from 25.9 to 42.1 µg/L (22.9 µg/g creatinine to 57.9 µg/g creatinine) for 5-HNMP and 31.4 to 45.8 µg/L (36.7 µg/g creatinine to 54.3 µg/g creatinine) for 2-HMSI. For the creatinine adjusted values we found a slight, but significant increase in 2-HMSI and 5-HNMP concentrations (both *p* < 0.001). The NEP metabolite 2-HESI was detected in the samples of all years with median concentrations between 2.5 and 38.4 µg/L (3.4 µg/g creatinine to 46.0 µg/g creatinine). For 5-HNEP median concentrations were all below LOQ except for 1999 with a median concentration of 9.7 µg/L (10.1 µg/g creatinine). For 5-HNEP more than 50% of the samples had concentrations < LOQ. Accordingly, 5-HNEP was excluded from time trend analysis. 2-HESI did not show any trend over the timeframe investigated. Thus, in this study with urine samples from 20 to 30 year old students from Münster, we could not observe any immediate effects of regulatory measures (starting in 2009 for NMP and 2013 for NEP) on general population exposure to the two alkyl pyrrolidones NMP and NEP. One possible explanation could be an increase in production volumes of products containing NMP or NEP, compensating the decrease in alkyl pyrrolidone concentrations. However, data on productions volumes of such products is not available so this remains speculative. Anyway, as already discussed above, even maximum exposures observed were and still are well below HBM I values (Human Biomonitoring assessment values) for single and combined NMP/NEP exposures.

**Table 5:** Medians and 95th percentiles of the NMP and NEP metabolite concentrations adjusted to creatinine

concentration (µg/g creatinine)	NMP				NEP			
	5-HNMP		2-HMSI		5-HNEP		2-HESI	
Year	Median	95th Percentile	Median	95th Percentile	Median	95th Percentile	Median	95th Percentile
1991	22.9	66.6	36.7	98.9	n.a.	272	26.1	329
1995	25.4	79.1	44.0	83.2	n.a.	205	4.6	272
1999	35.9	133	39.8	108	10.1	380	46.0	446
2003	29.1	80.1	40.2	92.8	n.a.	488	8.2	321
2006	43.2	111	47.3	116	n.a.	199	6.5	306
2008	38.1	101	46.8	134	n.a.	85.8	3.7	104
2010	57.9	115	53.8	113	n.a.	207	3.4	203
2012	37.7	109	48.6	106	n.a.	218	7.8	261
2014	52.9	202	54.3	131	n.a.	366	27.8	311

> 50% of concentrations (µg/L) below LOQ

*n.a*. no value available

Another observation to note is that, while median concentrations of the NEP metabolites were consistently below median concentrations of the respective NMP metabolites, the 95th percentiles of NEP metabolites were always higher (except for the year 2008). This would indicate to lower median NEP exposure, compared to NMP, but higher upper bound exposures to NEP, compared to NMP, which is also reflected in the highest hazard quotient found for NEP. Currently, it is still unclear, which sources are responsible for this NEP exposure, especially in samples pre 2008. We are not aware of any significant (industrial) NEP uses up to this time. We only found hints that NEP could be a side product in the synthesis of *N*-vinyl-2-pyrrolidone (Fink 2011, WO-Patent WO/2006/023118A1). Additionally, though the authors are not aware of any, a natural occurrence of NEP cannot be completely ruled out.

Finally, we calculated the daily intakes (DIs) for both NMP and NEP according to Eq. (1). As can be taken from Table 6, the DIs range between 1.6 µg/kg bw/day (1991, 95th percentile 4.6 µg/kg bw/day) and 2.9 µg/kg bw/day (2010, 95th percentile 7.6 µg/kg bw/day) for NMP and 0.1 µg/kg bw/day (2010, 18.2 µg/kg bw/day) and 2.3 µg/kg bw/day (1999, 95th percentile 27.9 µg/kg bw/day) for NEP. A slight increase in the DIs of NMP can be seen (*p* < 0.001), no trend was observable for NEP (*p* < 0.080). These results (based on 24-h urine concentrations, individual body weights and urinary metabolite conversion factors) are in line with the observations from the creatinine adjusted metabolite concentrations.

**Table 6:** Calculated median DIs (and 95th percentiles) for the compounds NMP and NEP by their respective metabolites 5-HNMP and 2-HMSI and accordingly 5-HNEP and 2-HESI for the investigated years

DI (µg/kg bw/day)	NMP		NEP	
Year	Median DI	95th Percentile	Median DI	95th Percentile
1991	1.6	4.6	1.1	23.1
1995	2.0	4.1	0.2	17.4
1999	2.0	4.5	2.3	27.9
2003	1.9	4.8	0.3	36.1
2006	2.4	6.2	0.2	11.9
2008	2.4	5.0	0.2	5.5
2010	2.9	7.6	0.1	18.2
2012	2.2	4.6	0.3	14.4
2014	2.7	7.6	1.1	18.4
all years	2.2	5.5	0.3	20.1

## Conclusion

With this study we were able to show that young adults of the German population have been continuously exposed to NMP and NEP at least since 1991, up to 2014. While exposure to NMP has been expected due to its wide field of (mainly industrial) applications, the ubiquitous exposure to NEP (previously marketed as a substitution product to NMP) came unexpected. For NEP, the sources of past exposures need to be unveiled. We did not observe an effect of regulatory measures enacted in recent years in the EU (2009–2013) on lowering alkyl pyrrolidone exposures in our study population. However, metabolite concentrations in all ESB samples were clearly below the HBM values for NMP and NEP derived by the German HBM Commission. A cumulative risk assessment by calculation of the hazard index (HI) based on the HBM I values of NMP and NEP as acceptable levels revealed a maximum HI of 0.09 which is clearly below 1, the level that would indicate a combined exposure above HBM I. Consequently, the results based on the ESB study population do not raise toxicological concerns towards the level of combined NMP and NEP exposure.

Ongoing measurements of 24-h urine samples of the ESB in the next years may investigate the possible effect of the regulatory measures imposed in the meantime on both alkyl pyrrolidones. In addition, study populations other than the ESB (20–30-year-old university students) should be investigated to broaden the database on exposure to alkyl pyrrolidones in the full German population, including susceptible subpopulations. Surveys such as GerES (German Environmental Survey) (Becker et al. 2007) could answer this question for other subpopulations like infants and children.

## Electronic supplementary material

Below is the link to the electronic supplementary material.

Supplementary material 1 (DOCX 462 KB)
