# Correction to: Metabolites of the alkyl pyrrolidone solvents NMP and NEP in 24-h urine samples of the German Environmental Specimen Bank from 1991 to 2014

**DOI:** 10.1007/s00420-019-01506-4

**Published:** 2019-12-18

**Authors:** Nadin Ulrich, Daniel Bury, Holger M. Koch, Maria Rüther, Till Weber, Heiko-Udo Käfferlein, Tobias Weiss, Thomas Brüning, Marike Kolossa-Gehring

**Affiliations:** 1 Institute for Prevention and Occupational Medicine of the German Social Accident Insurance, Institute of the Ruhr-Universität Bochum (IPA), Bürkle-de-la-Camp Platz 1, 44789 Bochum, Germany; 2 Department of Analytical Environmental Chemistry, Helmholtz-Centre for Environmental Research-UFZ, Permoserstr. 15, 04318 Leipzig, Germany; 3 German Environment Agency (UBA), Corrensplatz 1, 14195 Berlin, Germany

## Correction to: International Archives of Occupational and Environmental Health (2018) 91:1073–1082 10.1007/s00420-018-1347-y

The article Metabolites of the alkyl pyrrolidone solvents NMP and NEP in 24-h urine samples of the German Environmental Specimen Bank from 1991 to 2014, written by Nadin Ulrich, Daniel Bury, Holger M. Koch, Maria Rüther, Till Weber, Heiko-Udo Käfferlein, Tobias Weiss, Thomas Brüning, Marike Kolossa-Gehring, was originally published electronically on the publisher’s internet portal on 22 August 2018 without open access. With the author(s)’ decision to opt for Open Choice the copyright of the article changed on 12 December 2019 to © The Author(s) 2019 and the article is forthwith distributed under a Creative Commons Attribution 4.0 International License (https://creativecommons.org/licenses/by/4.0/), which permits use, sharing, adaptation, distribution and reproduction in any medium or format, as long as you give appropriate credit to the original author(s) and the source, provide a link to the Creative Commons licence, and indicate if changes were made.

The original article has been corrected.

