# Supplementary material for: Metabolites of the alkyl pyrrolidone solvents NMP and NEP in 24-h urine samples of the German Environmental Specimen Bank from 1991 to 2014
Source: Int Arch Occup Environ Health. 2018 Aug 22;91(8):1073–82. doi: 10.1007/s00420-018-1347-y (PMC6908567; doi:10.1007/s00420-018-1347-y)
Supplement: Supplementary file 1 — Supplementary material 1 (DOCX 462 KB) [file 420_2018_1347_MOESM1_ESM.docx]

**SI - Metabolites of the alkyl pyrrolidone solvents NMP and NEP in 24-h urine samples of the German Environmental Specimen Bank from 1991 to 2014**

Nadin Ulrich^1,2^, Daniel Bury^1^, Holger M. Koch^1^, Maria Rüther^3^, Till Weber^3^, Heiko-Udo Käfferlein^1^, Tobias Weiss^1^, Thomas Brüning^1^, Marike Kolossa-Gehring^3^

^1^Institute for Prevention and Occupational Medicine of the German Social Accident Insurance, Institute of the Ruhr-Universität Bochum (IPA), Bürkle-de-la-Camp Platz 1, 44789 Bochum, Germany

^2^Department of Analytical Environmental Chemistry, Helmholtz-Centre for Environmental Research - UFZ, Permoserstr. 15, 04318 Leipzig, Germany

^3^German Environment Agency (UBA), Corrensplatz 1, 14195 Berlin, Germany

Corresponding Author: Daniel Bury

e-mail: bury@ipa-dguv.de

tel: +49 – (0)234 302-4796

OrcidID:

Bury: 0000-0003-1283-3133

Koch: 0000-0002-8328-2837

Käfferlein: 0000-0001-5150-4979

Brüning: 0000-0001-9560-5464

**Content**

**SI 1 - Concentrations of the respective NMP and NEP metabolites for all years**

**SI 2 - Correlations of the NMP metabolites (5-HNMP and 2-HMSI) and NEP metabolites (5-HNEP and 2-HESI); in µg/L and µg/g creatinine**

**SI 3 - Median and 95^th^ percentile of the NMP and NEP metabolite concentrations for the investigated years.**

**SI 4 - Boxplots of the metabolites 5-HNMP, 5-HNEP, 2-HMSI, and 2-HESI (µg/L)**

**SI 1 - Concentrations of the respective NMP and NEP metabolites for all years**

|  | NMP | | NEP | |
| --- | --- | --- | --- | --- |
| concentration [µg/L] | 5-HNMP [µg/L] | 2-HMSI [µg/L] | 5-HNEP [µg/L] | 2-HESI [µg/L] |
| **all samples** | | | | |
| **median** | 30.3 | 38.8 | <LOQ | 6.1 |
| **geometric mean** | 29.1 | 38.0 | 2.8 | 8.8 |
| **95^th^percentile** | 98.1 | 100 | 212 | 230 |
| **min** | <LOQ | <LOQ | <LOQ | <LOQ |
| **max** | 655 | 358 | 962 | 950 |
| **n** | 540 | 540 | 540 | 540 |
| **n > LOQ** | 529 | 538 | 188 | 409 |
| **% > LOQ** | 98.0% | 99.6% | 34.8% | 75.7% |
| **male** | | | | |
| **median** | 29.8 | 40.2 | <LOQ | 6.4 |
| **geometric mean** | 27.8 | 40.4 | <LOQ | 8.0 |
| **95^th^percentile** | 96 | 114 | 152 | 190 |
| **min** | <LOQ | 6.7 | <LOQ | <LOQ |
| **max** | 655 | 358 | 962 | 826 |
| **n** | 270 | 270 | 270 | 270 |
| **n > LOQ** | 261 | 270 | 86 | 199 |
| **% > LOQ** | 96.7% | 100.0% | 31.9% | 73.7% |
| **female** | | | | |
| **median** | 30.3 | 36.9 | <LOQ | 5.7 |
| **geometric mean** | 30.4 | 35.8 | 3.3 | 9.5 |
| **95^th^percentile** | 100 | 89 | 228 | 266 |
| **min** | <LOQ | <LOQ | <LOQ | <LOQ |
| **max** | 242 | 213 | 526 | 950 |
| **n** | 270 | 270 | 270 | 270 |
| **n > LOQ** | 268 | 268 | 102 | 210 |
| **% > LOQ** | 99.3% | 99.3% | 37.8% | 77.8% |

**SI 2 Correlations of the NMP metabolites (5-HNMP and 2-HMSI) and NEP metabolites (5-HNEP and 2-HESI); in µg/L and µg/g creatinine**


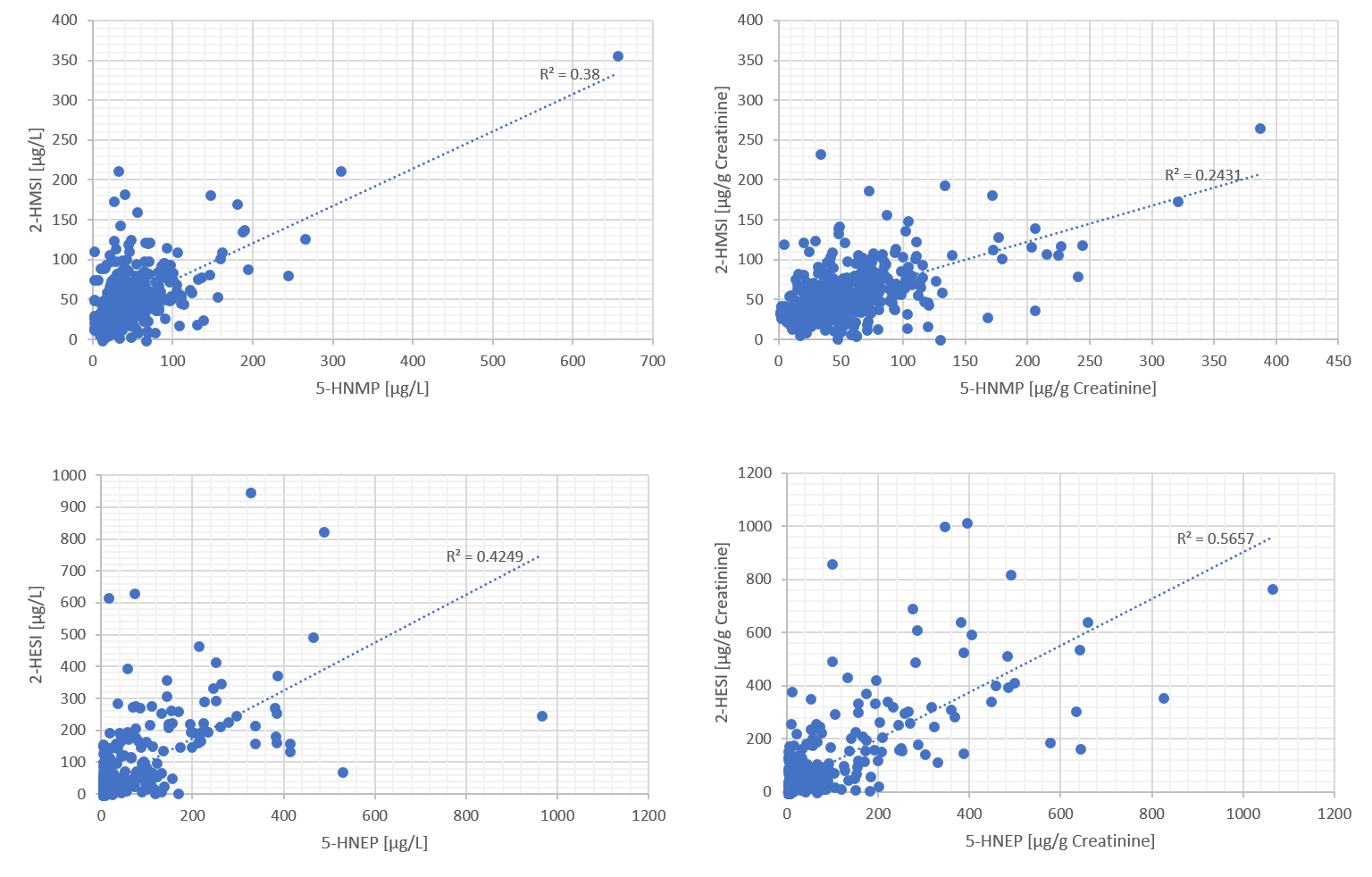


**SI 3 – Median and 95^th^ percentile of the NMP and NEP metabolite concentrations for the investigated years.**

| concentration [µg/L] | NMP | | | | NEP | | | |
| --- | --- | --- | --- | --- | --- | --- | --- | --- |
|  | 5-HNMP | | 2-HMSI | | 5-HNEP | | 2-HESI | |
| year | median | 95^th^ percentile | median | 95^th^ percentile | median | 95^th^ percentile | median | 95^th^ percentile |
| 1991 | 25.9 | 79.5 | 40.0 | 95.6 | <LOQ | 337 | 22.5 | 300 |
| 1995 | 27.8 | 92.2 | 45.8 | 121 | <LOQ | 194 | 6.7 | 282 |
| 1999 | 33.1 | 95.6 | 38.3 | 95.0 | 9.7 | 237 | 38.4 | 314 |
| 2003 | 26.5 | 83.4 | 39.1 | 101 | <LOQ | 248 | 8.5 | 182 |
| 2006 | 30.2 | 95.0 | 42.4 | 84.7 | <LOQ | 111 | 5.2 | 197 |
| 2008 | 27.6 | 79.0 | 35.8 | 98.4 | <LOQ | 70.0 | 2.5 | 70.7 |
| 2010 | 42.1 | 154 | 36.9 | 104 | <LOQ | 116 | 2.8 | 155 |
| 2012 | 26.2 | 62.4 | 31.4 | 73.5 | <LOQ | 133 | 4.0 | 215 |
| 2014 | 37.6 | 112 | 39.0 | 111 | <LOQ | 217 | 15.7 | 167 |

**SI 4 - Boxplots of the metabolites 5-HNMP, 5-HNEP, 2-HMSI, and 2-HESI (µg/L)**

**Fig. SI 4** Boxplots of the metabolites 5-HNMP, 5-HNEP, 2-HMSI, and 2-HESI for the investigated years. The concentrations are given in [µg/L], the average is indicated by the squared dot, the boxes show the 25^th^, 50^th^, and 75^th^ percentile and the whiskers the 5^th^ and 95^th^ percentile, respectively. The minimum and maximum concentrations are indicated by a dash.
